# Twenty years of ecosystem response after clearcutting and slashburning in conifer forests of central British Columbia, Canada

**DOI:** 10.1371/journal.pone.0172667

**Published:** 2017-02-24

**Authors:** Julia R. Chandler, Sybille Haeussler, Evelyn H. Hamilton, Michael Feller, Gary Bradfield, Suzanne W. Simard

**Affiliations:** 1 Department of Forest and Conservation Sciences, University of British Columbia, Vancouver, British Columbia, Canada; 2 Ecosystem Science and Management, University of Northern British Columbia, Prince George, British Columbia, Canada; 3 Centre for Livelihoods and Ecology, Royal Roads University, Victoria, British Columbia, Canada; 4 Department of Botany, University of British Columbia, Vancouver, British Columbia, Canada; Chinese Academy of Forestry, CHINA

## Abstract

Forests are being clearcut over extensive areas of western North America, but plant community response to harvesting and slashburning under varying climatic conditions in central British Columbia, Canada is still largely unknown. Evaluation of resilience is hampered by the short history of logging, lack of long-term experiments and methodological limitations. To test the effect of clearcut logging, prescribed burning and reforestation on forest resilience, we recorded vascular plant cover repeatedly after treatment between 1981 and 2008 in 16 permanent research installations in three biogeoclimatic zones: Engelmann Spruce-Subalpine Fir, Interior Cedar-Hemlock and Sub-Boreal Spruce. We created a plant-trait dataset for the 181 recorded species to define plant functional types representing groups of plants that behave in similar ways and/or produce similar ecological outcomes. These plant functional types, along with taxonomic analysis of diagnostic and indicator species, were then used to evaluate plant community response to disturbance. Twenty years post-treatment, species diversity increased in all zones and plant abundance was greatest in the Interior Cedar-Hemlock. Cover of understory plant functional types associated with mature conifer forests increased in all zones, constituting a significant proportion (> 40%) of the vegetation community by year 20. Response patterns varied by zone and with time. Understory species diagnostic of mature forests were present in all zones by year 20, but we identified indicator species sensitive to slashburning or requiring more time for recovery, including white-flowered rhododendron (*Rhododendron albiflorum*) and devil's club (*Oplopanax horridus*). Overall, loss of compositional or functional diversity following harvest and site remediation was not detected, suggesting that montane and subalpine forests in British Columbia are resilient to this treatment. However, because these forests can be slow to recover from disturbance, the post-disturbance assessment window of this study may not have been long enough to detect diminishment of ecosystem resilience.

## Introduction

There is considerable interest in assessing the resilience of forests to management strategies and natural disturbance in the face of changing climates and evolving management practices [1–3]. While many definitions of resilience are used [4, 5], and several theories and strategies to maintain it have been proposed [1–3, 6], there is widespread consensus that resilience in forest ecosystems depends upon multiple factors, including plant life-history traits, disturbance characteristics, climate sensitivity, and successional dynamics [6–8].

North American forest plant communities have been shown to be quite resilient to management of forests for timber [8] and to fire [9]; even fires of fairly high severity [10, 11]. Yet, some studies suggest a high degree of site-specificity in resilience [8], and that resilience varies with both the treatment [9–12] and the response variables measured [13]. Moreover, the timeframe over which resilience of these forests has been commonly assessed (i.e., < 20 years) may not be sufficiently long to provide reliable conclusions [10, 12].

Grouping plants according to shared traits and functional characteristics, hereafter plant functional types (PFTs) [14], has been a very successful approach for modeling the response of forest communities and species to disturbance [7, 13, 15–17] and for generalizing plant response across phytogeographic gradients. Specifically, traits that confer resilience (e.g. resprouting ability, seed dormancy and dispersal) were important determinants of community response in fire prone ecosystems in Australia [7]; proportions of plants with different traits were related to fire frequency and climatic conditions. Models that explain the proportions of resprouting versus seedbanking life history types include gap-dependent recruitment [18, 19], disturbance frequency and type [20, 21], and resource/productivity [22].

Because each plant species has adapted to a specific range of environmental conditions (niche), plant species presence can also be a useful indicator that provides a lens into site quality based on the knowledge of plant-environment relationships [23]. Indicator species response can be very useful in characterizing site-level properties across gradients of disturbance intensity [24, 25], climate [7, 15] and soil productivity [26], as well as representing key traits of functional groups [15, 24, 25].

Recent studies of understory response to disturbance in eastern North American boreal forests include indicator species analysis and plant functional type (PFT) assessments. This combined approach retains site-level detail while also providing further insights into ecosystem resilience by reducing the functional redundancy within ecosystems and allowing for generalizations across sites. Such studies have found, for example, that compositional and functional attributes of planted stands require more time to develop after harvest and reforestation compared to naturally regenerating stands [27]; and that habitat characteristics explained more than three times the variation in boreal plant community composition than variation in fire severity [13].

Our study is set in west central British Columbia (BC), Canada, where fires in conifer-dominated landscapes have historically been more frequent and more severe than those of the eastern boreal mixedwoods [28]. For most of the 20^th^ Century, prescribed burning (specifically slashburning) has been widely used after timber harvest as a means of reducing fire hazard, costs of site preparation, and competing vegetation [29–31], and there has been recent impetus to expand this practice [32]. While ecosystem models have been developed to predict the effect of harvesting and/or fire on long-term tree biomass production [33] and stand development [34, 35], few studies have examined long-term response of understory plant communities after clearcutting and slashburning in BC [8, 9].

BC has exceptionally varied geography and ecosystems [36]. Thus, while resilience is known to vary according to ecosystem characteristics [37], it is difficult to extrapolate results from other geographic regions such as the eastern boreal forest [13], the U.S. Rocky Mountains [11] and Pacific coastal forests [38, 39], to those in central BC. Slow understory redevelopment has, however, been reported following logging without slashburning in subalpine forests of central BC [40].

In the current study, repeated vegetation abundance measures were collected over a period of 22 years after clearcut logging, slashburning and reforestation in three montane to subalpine forest zones in central BC. There were 16 permanent research sites with known ecosystem classification, pre-harvesting conditions, treatment history, and fire severity. Our objectives were to: (1) develop a comprehensive set of PFTs for the vascular plants recorded in the study sites to be used in this and future studies; and (2) test the hypothesis, using both PFTs and taxonomic (indicator and diagnostic species) level measurements, that the climatic and environmental gradients presented by the zones and study sites would influence understory vascular plant resilience to the forestry treatment.

## Materials and methods

### Study site descriptions

**Fig 1:**
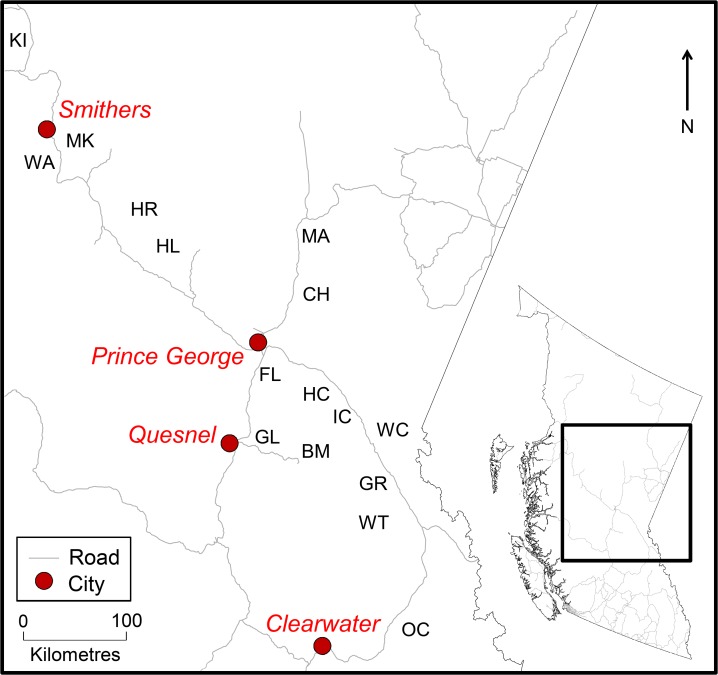
Map of study site locations in central British Columbia. Study site abbreviations: BM = Brinks Mill, CH = Chuchinka Creek, FL = Francis Lake, GL = Genevieve Lake, GR = Goat River, HC = Haggen Creek, HL = Helene, HR = Herron, IC = Indianpoint Creek, KI = Kinskuch, MA = Mackenzie, MK = McKendrick, OC = Otter Creek, WA = Walcott, WC = Walker Creek, WT = West Twin.

This research was carried out on public land in established research experimental sites in cooperation with the BC Ministry of Forests, Lands and Natural Resource Operations; no special permission was required. This field study did not involve endangered or protected species. Study sites included 16 long-term research installations (Table 1) maintained by the BC Forest Service and located in central interior BC, Canada (Fig 1). The 16 study sites occur in the Engelmann Spruce-Subalpine Fir (ESSF), Interior Cedar-Hemlock (ICH), and Sub-Boreal Spruce (SBS) biogeoclimatic zones [41]; see S1 Table for site classification details.

**Table 1:** Study site classifications, climate, treatments, burn impact and sampling history.

Site	BEC subzone^a^	MAT^b^	MAP^b^	Year Logged	Year Burned	LFH^c^	DOB^d^ (mean)	DOB^d^ (se)	%LFH^e^ (mean)	%LFH^e^ (se)	*n*^f^	Species Planted^g^	Years Sampled^h^	*n*^i^
Herron*	ESSFmc	1.6	644	1982	1983	9.8	1.3	0.2	18.0	4.0	36	Pl	-1,1,2,3,4,8,17	10
McKendrick	ESSFmc	1.9	653	1984	1985	7.9	2.3	0.2	32.0	2.6	90	Pl	-1,1,2,3,4,5,7	15
Otter Creek^z^*	ESSFwc	1.3	1391	1987	1989	4.1	1.0	0.1	24.0	1.4	359	Sx, Pl, Bl	-1,1,2,3,5,11,18	34
West Twin	ESSFwk	2.3	955	1987	1989	6.0	3.1	0.2	54.0	1.5	571	Sx	-1,1,2,3,5,11	30
Kinskuch	ICHmc	5.3	1031	1981	1982	5.3	2.9	0.3	53.0	6.3	48	Pl	1,2,3,4,5,7,10	30
Walker Creek*	ICHvk	3.0	1064	1985	1986	11.5	2.0	0.2	20.0	1.7	512	Sx	-1,1,2,3,5,10,21,22	120
Goat River*	ICHwk	3.1	1057	1987	1988	6.1	1.1	0.1	18.0	2.1	126	Sx	-1,1,2,3,5,10,20	6
Helene*	SBSmc	2.7	608	1981	1982	8.7	2.4	0.2	37.0	6.0	36	Pl	1,2,3,4,5,7,10,18,21	5
Walcott*	SBSmc	3.0	588	1981	1982	10.4	3.7	0.3	37.0	2.8	36	Pl	1,2,3,4,5,7,10,16,21	5
Francis Lake*	SBSmk	3.4	686	1985	1986	12.5	3.8	0.4	30.0	1.8	90	Sx, Pl	1,2,3,5,10,21	6
Genevieve Lake*	SBSmk	3.4	651	1985	1986	7.0	1.8	0.3	25.0	2.7	220	Sx, Pl	1,2,3,5,10,21	4
Brinks Mill*	SBSvk	3.6	704	1985	1986	7.7	2.1	0.5	28.0	0.4	60	Sx	1,2,3,5,10,21	3
Haggen Creek	SBSvk	3.2	798	1983	1986	8.1	3.2	0.4	40.0	4.0	60	Sx	1,2,3,5	8
Indianpoint Creek	SBSvk	3.6	724	1985	1986	6.4	0.6	0.1	9.0	1.2	60	Sx	1,2,3,5,10	3
Chuchinka	SBSwk	2.8	851	1986	1987	8.9	1.3	0.1	17.0	1.0	344	Sx	-1,1,2,4,5,10	29
Mackenzie*	SBSwk	2.7	755	1987	1988	12.7	3.1	0.3	25.0	1.8	84	Sx	-1,1,2,3,5,10,20	6

^a^ Derived from the BEC system (m = moist, w = wet, v = very wet; k = cool, c = cold) [41].

^b^ Mean annual temperature (°C) and mean annual precipitation (mm) averaged for 1980–2002 [42, 43].

^c^ LFH—pre-burn depth (cm) of Litter, Fermented and Humic layer of the forest floor including woody debris <1 cm in diameter.

^d^ DOB—mean depth (cm) of burn of forest floor (LFH) layers.

^e^ Percent consumption of LFH layer.

^f^ Number of DOB pins measured.

^g^ Tree species planted: Sx = *P*. *glauca* x *engelmannii*, Bl = *A*. *lasiocarpa*, Pl = *P*. *contorta* var. *latifolia*.

^h^ Measurements collected after clearcutting but before prescribed burn are indicated by -1; 1–22 indicates growing seasons after prescribed burn. Vegetation monitoring quadrats were 5 m x 5 m with the exceptions of Otter Creek (3 m x 3 m quadrats) and Walker Creek (1 m diameter circular plots).

^i^ Number of vegetation plots.

^z^ Trees were not planted in the vegetation monitoring quadrats at this site but were planted elsewhere on the site. Quadrats were distributed throughout the site.

^*^ Subset of 10 sites with > 17 years of vegetation data.

The ESSF is a subalpine forest zone with a severe climate characterized by a long cold winter and a short cool summer; Engelmann spruce (*Picea engelmannii*) and subalpine fir (*Abies lasiocarpa)* are dominant in mature and old growth forests, and lodgepole pine (*Pinus contorta* var. *latifolia*) is a pioneer after disturbance in drier warmer ESSF forests. Understories are dominated by ericaceous shrubs, rhizomatous forbs Sitka valerian (*Valeriana sitchensis*) and *Arnica* spp., and a mix of mosses and leafy liverworts. The ICH is characterized by cool, wet winters and warm, relatively dry summers; it is the most productive interior zone with the greatest diversity of tree species, including western hemlock (*Tsuga heterophylla*), western redcedar (*Thuja plicata*) and, in moist areas, hybrid white spruce (*Picea glauca* x *engelmannii)*. Interior Douglas-fir (*Pseudotsuga menziesii* var. *glauca*) and lodgepole pine occur in drier warmer parts of the zone. Black huckleberry (*Vaccinium membranaceum*), thimbleberry (*Rubus parviflorus*), queen's cup (*Clintonia uniflora*) and feathermosses are common in the understory. The SBS occurs on gently rolling plateaus and is generally drier and warmer than the ESSF and drier and colder than the ICH; *P*. *glauca* x *engelmannii* and *A*. *lasiocarpa* are the dominant species, interspersed with abundant wetlands and large expanses of seral *P*. *contorta* var. *latifolia* and trembling aspen (*Populus tremuloides*) forest resulting from past wildfires. Understory is typically dominated by black twinberry (*Lonicera involucrata*), highbush-cranberry (*Viburnum edule*), birch-leaved spirea (*Spiraea betulifolia*), bunchberry (*Cornus canadensis*) and feathermosses [41]. Climate variables (MAT and MAP) for the study sites were derived from 1980 to 2002 using the ClimateBC model [42, 43].

### Field procedures

All sites were clearcut between 1981 and 1987 (Table 1); harvesting was done in the winter to help protect the forest floor and understory vegetation. Within three years of logging, all sites were slashburned in the fall (except Otter Creek, which had blocks burned in spring and fall) and then planted with *P*. *glauca* x *engelmannii*, *P*. *contorta* var. *latifolia* and/or *A*. *lasiocarpa* seedlings. To measure fire severity, depth-of-burn (DOB) pins were inserted into the soil, and forest floor (LFH) depths were measured prior to and immediately after the burn [44]. DOB was calculated as the difference between the pre-fire and post-fire measurements. After slashburning, percent cover and height of each vascular plant species were visually estimated in each permanent plot; the sum of percent cover could exceed 100%. Plot size and number of plots varied by site. Vegetation was re-sampled, generally 1, 2, 3, 5, 10, and 20 years following slashburning for most sites. Detailed field methods [45] and ten-year vegetation response summaries were previously reported [46–54].

### Statistical analyses

Cluster analysis was performed to group each of the 181 vascular plant species measured on the 16 sites into one of eight PFTs. A species-trait dataset (S2 Table) was created that included traits selected from the literature (S1 Appendix) to differentiate strategies, requirements, and responses of individual species related to logging and slashburning. This provides an extensive reference for vascular plants of central BC for this and future studies. Plant attributes used in the cluster analysis included physical properties (plant height, plant duration, leaf duration, N-fixation), seed properties (seed quantity, seed dispersal, seed longevity, seed size), root properties (depth of rooting, vigour of sprouting, rate of lateral spread, dominant mycorrhizal guild), preference of ground surface material, and light index; all traits were scaled to 1. Creating PFTs reduced the many individual species recorded in our study sites to only a few groups that represent ecological functions conferring resilience to disturbance [55].

A subset of 10 study sites had >16 years of post-treatment measurements; these data were used for the comparisons and statistical analyses described below. The ten sites varied in the number of years post-burn data was collected, the number of plots per site, and climate (Table 1).

British Columbia developed a Biogeoclimatic Ecosystem Classification (BEC) system to serve as a framework for resource management [56] and designed it to enable users to classify an ecosystem based on species composition and soil properties, regardless of seral stage. The BEC system combines climatic (or zonal) and ecosystem level classifications. Biogeoclimatic units (e.g., zones, subzones) were mapped and ecosystems within these units (e.g. site series) identified. Ecosystems are differentiated using a “diagnostic combinations of species” (DCS). These species are well adapted to environmental conditions typical of a biogeoclimatic unit, include vascular plants (i.e. trees and understory plants) and nonvascular plants, and had > 40% presence and > 10% cover in sampled plots [57].

We used DCS at the BEC zonal level to evaluate the extent to which ecosystems recovered to the floristic composition typical of the biogeoclimatic zone. We focused on the subset of these species that are diagnostic understory vascular species, hereafter diagnostic species, in our analysis since tree species were planted on the sites and their presence does not reflect natural regeneration processes. Species constancy in each zone was determined by the number of sites in which a diagnostic species occurred at year 20 compared to the number of sites for which a species was diagnostic; and the presence of each diagnostic species was noted by cover class (< 0.1%, 0.1–1%, 1.1–5%, 5.1–10%, 10.1–25%, and 25.1–100%).

Indicator species analysis was used to assess the strength of the correlations between the presence of vascular plant species and the three biogeoclimatic zones. There were 2 sites in the ESSF (*n* = 44 plots), 2 sites in the ICH (*n* = 136 plots) and 6 sites in the SBS (*n* = 49 plots). Indicator species for each zone were identified by calculating the indicator value index (which includes a correction for unequal group sizes) [58] based on repeated measurements of vascular plant species cover at the plot scale (*n* = 229 plots) at year 1 (80 species), year 5 (91 species), year 10 (115 species), and year 20 (133 species). Species with indicator values > 0.5 (*p* < 0.05) were considered ‘moderate indicators’; and species with indicator values > 0.75 (*p* < 0.05) were considered ‘strong indicators’ of a zone.

The indicator value is the product of two components, called 'A' and 'B' [58, 59]. Component A (also known as *specificity*) is the probability that the surveyed site belongs to the target site group given the fact that the species has been found. Component B (also known as *fidelity*) is the probability of finding the species in sites belonging to the site group. Species with component values of ‘1’ were considered to have ‘complete’ specificity and/or fidelity.

To test for differences in cover of PFTs and indicator species over time, Friedman two-way ranked sum tests [60] were performed on repeated measures (1, 5, 10 and 20 years after treatment) in each zone, followed by Nemenyi post-hoc pairwise comparisons [61]. To test for differences in traits between PFTs and differences in PFT cover between zones, Kruskal-Wallis rank sum tests [62] with Chi-squared approximation for independent samples were performed, followed by Conover-Iman post-hoc multiple comparisons [63] and *p*-value adjustment with the Bonferroni correction for multiple comparisons. Significance tests were performed on the subset of 10 sites (with site used as a blocking factor): 2 sites in the ESSF (*n* = 44 plots); 2 sites in the ICH (*n* = 136 plots); and 6 sites in the SBS (*n* = 49 plots).

Statistical analyses were performed using the programming language R [64]. Cluster analysis was performed using the R package ‘vegan’ [65] with the hclust() function ‘ward.d2’ option [66, 67] applied to a Bray-Curtis [68] distance matrix created with the vegdist() function. The indicator species analysis was performed using the R package ‘indicspecies’ [69] with the multipatt() function (‘Inval.g’ and ‘duleg’ options). Friedman tests were performed with the friedman.test() function and post-hoc tests were performed using the R package ‘PMCMR’ [70].

## Results

### Grouping species by PFTs

**Fig 2:**
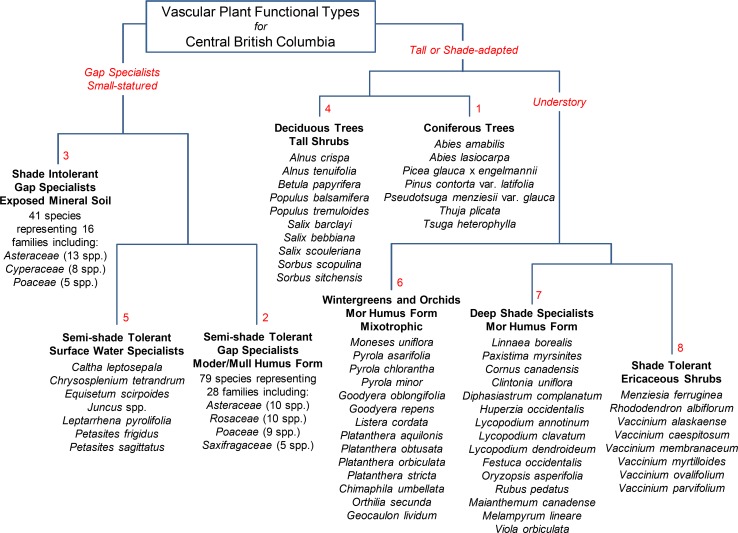
Plant functional types.

The cluster analysis grouped all species into eight PFTs (Fig 2). The analysis identified groups of plants that were either (a) tall or adapted to a shaded environment, or (b) small statured and light-requiring. The tall or shade-adapted species included coniferous trees (PFT 1; 7 species), deciduous trees and tall shrubs (PFT 4; 10 species), wintergreens and orchids (PFT 6; 14 species), small deep shade specialists (PFT 7; 15 species), and ericaceous shrubs (PFT 8; 8 species). The small statured and light-requiring species included semi-shade tolerant gap specialists (PFT 2; 79 species), shade intolerant gap specialists (PFT 3; 41 species), and semi-shade tolerant surface water specialists (PFT 5; 7 species) (S1 Fig).

### PFTs in the ESSF, ICH and SBS zones

**Fig 3:**
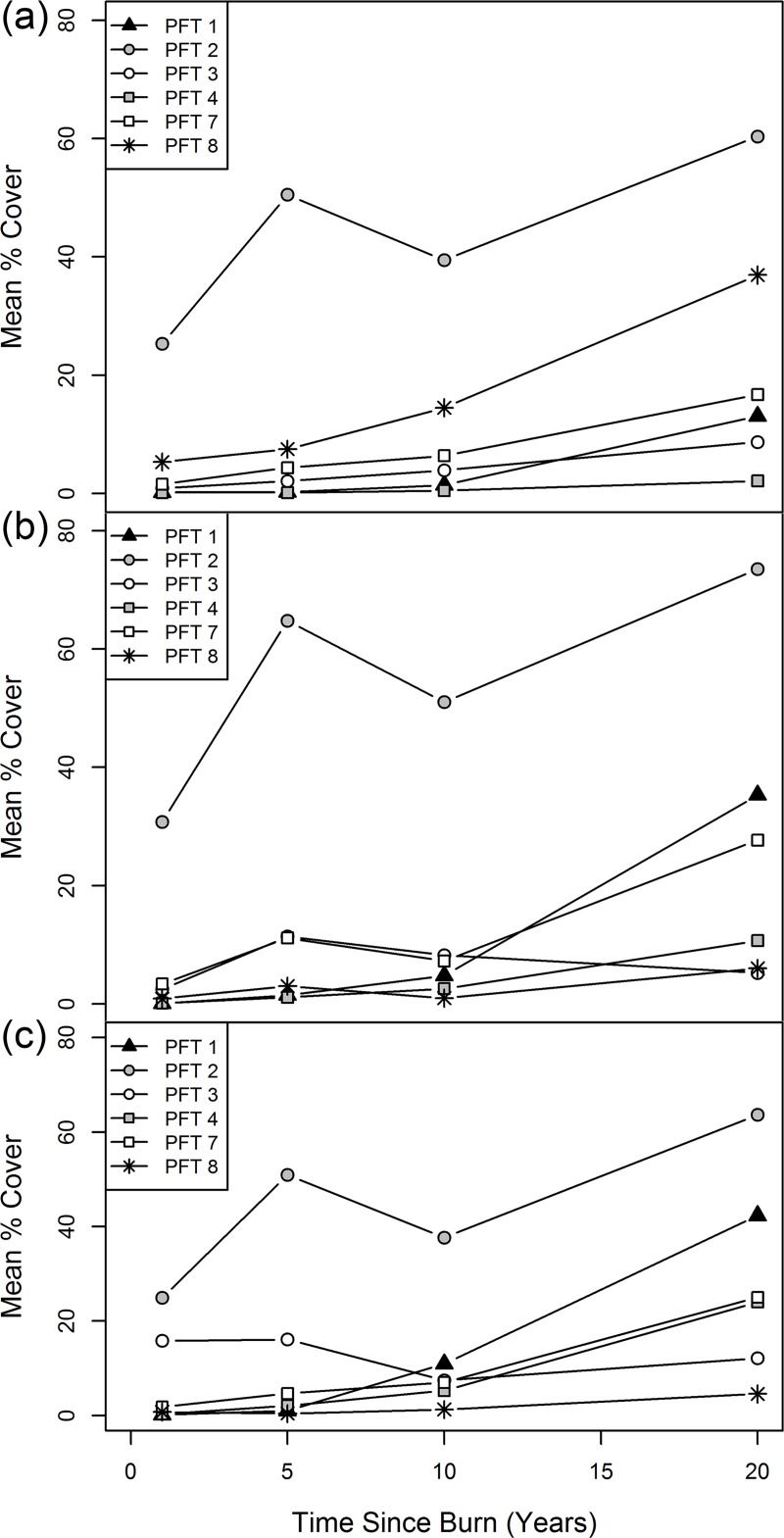
PFT abundance over time. Mean percent cover of PFTs over 20 years after clearcutting and slashburning in the ESSF (a), ICH (b) and SBS (c) zones. PFTs 5 and 6 (< 2% mean cover in any year) are not included.

PFT response (mean % cover) over time (Table 2, Fig 3) and between zones (Table 3) was as follows:

**Table 2:** PFT abundance over time. Repeated measurements of percent cover 1, 5, 10, and 20 years after clearcutting and slashburning in ESSF (2 sites; *n* = 44 plots), ICH (2 sites; *n* = 136 plots), and SBS (6 sites; *n* = 49 plots) zones. Friedman ranked sum tests with Nemenyi post-hoc pairwise multiple comparison test; ^*a*, *b*, *c*^ indicate significant differences between years (*p* < 0.05) for each PFT.

		Year 1			Year 5			Year 10			Year 20			Friedman chi-squared	
		mean	se	^* *^	mean	se	^* *^	mean	se	^* *^	mean	se	^* *^		*p*-value
**ESSF**															
1	Conifers	0.2	(0.2)	^*a*^	0.2	(0.1)	^*a*^	1.4	(0.3)	^*b*^	13.1	(8.2)	^*c*^	91.7	<0.001
2	Semi-shade tolerant gap specialists	25.3	(15.9)	^*a*^	50.5	(32.1)	^*b*^	39.5	(22.5)	^*b*^	60.4	(37.1)	^*b*^	46.8	<0.001
3	Shade intolerant gap specialists	0.9	(0.9)	^*a*^	2.1	(1.0)	^*b*^	3.9	(0.4)	^*b*^	8.7	(2.1)	^*b*^	37.9	<0.001
4	Deciduous trees and tall shrubs	0.1	(0.0)	^*a*^	0.1	(0.0)	^*a*^	0.5	(0.1)	^*a*^	2.1	(1.3)	^*a*^	13.3	0.004
5	Surface water specialists	0.0	(0.0)	^*NA*^	0.0	(0.0)	^*NA*^	0.0	(0.0)	^*NA*^	0.0	(0.0)	^*NA*^	NA	NA
6	Wintergreens and orchids	0.2	(0.1)	^*a*^	0.1	(0.0)	^*a*^	0.1	(0.1)	^*a*^	1.5	(0.9)	^*a*^	16.8	<0.001
7	Deep shade specialists	1.5	(0.2)	^*a*^	4.3	(0.2)	^*b*^	6.3	(2.2)	^*b*^	16.7	(9.9)	^*b*^	65.1	<0.001
8	Ericaceous shrubs	5.3	(2.9)	^*a*^	7.5	(4.0)	^*b*^	14.5	(10.4)	^*c*^	36.9	(22.0)	^*d*^	110.2	<0.001
**ICH**															
1	Conifers	0.1	(0.1)	^*a*^	1.5	(1.2)	^*a*^	4.8	(1.2)	^*b*^	35.3	(8.4)	^*c*^	249.5	<0.001
2	Semi-shade tolerant gap specialists	30.8	(28.4)	^*a*^	64.8	(39.7)	^*b*^	51.0	(43.0)	^*b*^	73.6	(51.7)	^*c*^	163.6	<0.001
3	Shade intolerant gap specialists	2.5	(1.7)	^*a*^	11.4	(3.4)	^*b*^	8.2	(4.3)	^*a*^	5.2	(3.2)	^*c*^	148.8	<0.001
4	Deciduous trees and tall shrubs	0.1	(0.1)	^*a*^	1.1	(0.5)	^*a*^	2.5	(1.7)	^*a*^	10.7	(6.5)	^*b*^	126.0	<0.001
5	Surface water specialists	0.0	(0.0)	^*a*^	0.0	(0.0)	^*a*^	0.0	(0.0)	^*a*^	0.0	(0.0)	^*a*^	6.0	0.116
6	Wintergreens and orchids	0.0	(0.0)	^*a*^	0.0	(0.0)	^*a*^	0.0	(0.0)	^*a*^	0.1	(0.1)	^*a*^	20.2	<0.001
7	Deep shade specialists	3.4	(2.9)	^*a*^	11.1	(4.3)	^*b*^	7.2	(3.3)	^*b*^	27.7	(17.8)	^*c*^	132.1	<0.001
8	Ericaceous shrubs	0.9	(0.6)	^*a*^	3.0	(1.4)	^*a*^	1.0	(0.8)	^*a*^	6.0	(5.4)	^*b*^	82.0	<0.001
**SBS**															
1	Conifers	0.2	(0.1)	^*a*^	1.0	(0.3)	^*a*^	11.0	(1.5)	^*b*^	42.3	(3.5)	^*c*^	133.3	<0.001
2	Semi-shade tolerant gap specialists	24.9	(10.8)	^*a*^	50.9	(14.7)	^*b*^	37.7	(8.6)	^*b*^	63.6	(20.7)	^*b*^	55.5	<0.001
3	Shade intolerant gap specialists	15.8	(4.0)	^*ab*^	16.1	(4.2)	^*a*^	7.5	(1.9)	^*b*^	12.2	(6.4)	^*b*^	14.3	0.003
4	Deciduous trees and tall shrubs	0.3	(0.1)	^*a*^	2.1	(0.5)	^*a*^	5.3	(1.3)	^*b*^	24.0	(7.6)	^*b*^	89.9	<0.001
5	Surface water specialists	0.0	(0.0)	^*a*^	0.1	(0.1)	^*a*^	0.1	(0.1)	^*a*^	0.1	(0.0)	^*a*^	6.9	<0.001
6	Wintergreens and orchids	0.0	(0.0)	^*a*^	0.0	(0.0)	^*a*^	0.1	(0.1)	^*a*^	1.7	(0.7)	^*b*^	57.5	<0.001
7	Deep shade specialists	1.8	(0.6)	^*a*^	4.7	(2.1)	^*a*^	7.0	(2.3)	^*b*^	25.0	(4.6)	^*c*^	92.2	<0.001
8	Ericaceous shrubs	0.8	(0.4)	^*ab*^	0.4	(0.2)	^*a*^	1.3	(0.8)	^*b*^	4.6	(2.5)	^*c*^	59.4	<0.001

**Table 3:** PFT abundance in the ESSF, ICH and SBS zones. Mean percent cover of PFTs 1, 5, 10, and 20 years after clearcutting and slashburning. Kruskal-Wallis ranked sum tests with Conover-Iman post-hoc pairwise multiple comparison test for independent samples and Bonferroni correction; ^*a*, *b*, *c*^ indicate significant differences between zones (*p* < 0.05) for each PFT.

		ESSF			ICH			SBS			Kruskal-Wallis chi-squared	
		mean	se	^* *^	mean	se	^* *^	mean	se	^* *^		*p*-value
**Year 1**												** **
1	Conifers	0.2	(0.2)	^*a*^	0.1	(0.1)	^*a*^	0.2	(0.1)	^*b*^	25.8	<0.001
2	Semi-shade tolerant gap specialists	25.3	(15.9)	^*a*^	30.8	(28.4)	^*b*^	24.9	(10.8)	^*c*^	130.6	<0.001
3	Shade intolerant gap specialists	0.9	(0.9)	^*a*^	2.5	(1.7)	^*b*^	15.8	(4.0)	^*c*^	143.0	<0.001
4	Deciduous trees and tall shrubs	0.1	(0.0)	^*a*^	0.1	(0.1)	^*a*^	0.3	(0.1)	^*b*^	14.0	<0.001
5	Surface water specialists	0.0	(0.0)	^*NA*^	0.0	(0.0)	^*NA*^	0.0	(0.0)	^*NA*^	NA	NA
6	Wintergreens and orchids	0.2	(0.1)	^*NA*^	0.0	(0.0)	^*NA*^	0.0	(0.0)	^*NA*^	NA	NA
7	Deep shade specialists	1.5	(0.2)	^*a*^	3.4	(2.9)	^*b*^	1.8	(0.6)	^*a*^	131.6	<0.001
8	Ericaceous shrubs	5.3	(2.9)	^*a*^	0.9	(0.6)	^*b*^	0.8	(0.4)	^*c*^	61.3	<0.001
**Year 5**				^* *^			^* *^			^* *^		
1	Conifers	0.2	(0.1)	^*a*^	1.5	(1.2)	^*a*^	1.0	(0.3)	^*b*^	225.1	<0.001
2	Semi-shade tolerant gap specialists	50.5	(32.1)	^*a*^	64.8	(39.7)	^*b*^	50.9	(14.7)	^*c*^	130.6	<0.001
3	Shade intolerant gap specialists	2.1	(1.0)	^*a*^	11.4	(3.4)	^*b*^	16.1	(4.2)	^*b*^	24.2	<0.001
4	Deciduous trees and tall shrubs	0.1	(0.0)	^*a*^	1.1	(0.5)	^*a*^	2.1	(0.5)	^*b*^	123.7	<0.001
5	Surface water specialists	0.0	(0.0)	^*NA*^	0.0	(0.0)	^*NA*^	0.1	(0.1)	^*NA*^	NA	NA
6	Wintergreens and orchids	0.1	(0.0)	^*NA*^	0.0	(0.0)	^*NA*^	0.0	(0.0)	^*NA*^	NA	NA
7	Deep shade specialists	4.3	(0.2)	^*a*^	11.1	(4.3)	^*b*^	4.7	(2.1)	^*a*^	82.7	<0.001
8	Ericaceous shrubs	7.5	(4.0)	^*a*^	3.0	(1.4)	^*b*^	0.4	(0.2)	^*c*^	225.0	<0.001
**Year 10**				^* *^			^* *^			^* *^		
1	Conifers	1.4	(0.3)	^*a*^	4.8	(1.2)	^*a*^	11.0	(1.5)	^*b*^	26.7	<0.001
2	Semi-shade tolerant gap specialists	39.5	(22.5)	^*a*^	51.0	(43.0)	^*b*^	37.7	(8.6)	^*c*^	130.6	<0.001
3	Shade intolerant gap specialists	3.9	(0.4)	^*a*^	8.2	(4.3)	^*a*^	7.5	(1.9)	^*a*^	75.6	<0.001
4	Deciduous trees and tall shrubs	0.5	(0.1)	^*a*^	2.5	(1.7)	^*a*^	5.3	(1.3)	^*b*^	109.4	<0.001
5	Surface water specialists	0.0	(0.0)	^*a*^	0.0	(0.0)	^*a*^	0.1	(0.1)	^*b*^	136.8	<0.001
6	Wintergreens and orchids	0.1	(0.1)	^*a*^	0.0	(0.0)	^*b*^	0.1	(0.1)	^*a*^	71.2	<0.001
7	Deep shade specialists	6.3	(2.2)	^*ab*^	7.2	(3.3)	^*b*^	7.0	(2.3)	^*a*^	44.6	<0.001
8	Ericaceous shrubs	14.5	(10.4)	^*a*^	1.0	(0.8)	^*b*^	1.3	(0.8)	^*c*^	17.3	<0.001
**Year 20**				^* *^			^* *^			^* *^		
1	Conifers	13.1	(8.2)	^*a*^	35.3	(8.4)	^*b*^	42.3	(3.5)	^*c*^	9.9	<0.001
2	Semi-shade tolerant gap specialists	60.4	(37.1)	^*a*^	73.6	(51.7)	^*b*^	63.6	(20.7)	^*c*^	130.6	<0.001
3	Shade intolerant gap specialists	8.7	(2.1)	^*a*^	5.2	(3.2)	^*b*^	12.2	(6.4)	^*a*^	30.5	<0.001
4	Deciduous trees and tall shrubs	2.1	(1.3)	^*a*^	10.7	(6.5)	^*b*^	24.0	(7.6)	^*c*^	95.4	<0.001
5	Surface water specialists	0.0	(0.0)	^*NA*^	0.0	(0.0)	^*NA*^	0.1	(0.0)	^*NA*^	NA	NA
6	Wintergreens and orchids	1.5	(0.9)	^*a*^	0.1	(0.1)	^*b*^	1.7	(0.7)	^*c*^	76.5	<0.001
7	Deep shade specialists	16.7	(9.9)	^*a*^	27.7	(17.8)	^*a*^	25.0	(4.6)	^*b*^	131.6	<0.001
8	Ericaceous shrubs	36.9	(22.0)	^*a*^	6.0	(5.4)	^*b*^	4.6	(2.5)	^*b*^	12.7	<0.001

***PFT 1****—Conifers*. Cover of PFT 1 was similar in all three zones in year 1 (mean < 0.2%), increased over time in all zones, and was greatest in SBS by year 20 (42%) followed by the ICH (35%) and the ESSF (13%).
***PFT 2****—Semi-shade tolerant gap specialists*. PFT 2 had the greatest number of species (79), had higher cover than any other PFT in all zones in comparable years, and had higher cover than the combined cover of all other PFTs in all zones until year 10. In all zones the trend in cover of PFT 2 was very similar, increasing between years 1 and 5, decreasing between years 5 and 10, and then increasing again between years 10 and 20.
***PFT 3****—Shade intolerant gap specialists*. Trends in cover for PFT 3 were different for each zone. Cover in the ESSF increased gradually to a maximum of 8.7% in year 20; cover in the ICH peaked at year 5 (11.4%) and then declined; and cover in the SBS was highest (approximately16%) in years 1 and 5 and then declined.
***PFT 4****—Deciduous trees and tall shrubs*. Cover of PFT 4 was low until after year 10 when it increased considerably. By year 20, PFT 4 species formed a significant part of the plant community in the ICH and SBS. *P*. *tremuloides* and paper birch (*Betula papyrifera*) were the dominant PFT 4 species.
***PFT 5****—Semi-shade tolerant surface water specialists*. PFT 5 was a minor component of the vegetation community, with the lowest cover of all PFTs (< 0.2% in any year). This PFT was not evident in the ESSF sites.
***PFT 6****—Wintergreens and orchids*. PFT 6 was a minor component in these zones. Cover of these species was < 2% in any year sampled and showed little change over time.
***PFT 7****—Deep shade specialists*. Cover of PFT 7 increased over time in all zones. In year 1 cover was between 1.5 and 3.4% and by year 20 it was between 16 and 27%, ranking third amongst PFTs in terms of cover. Characteristic species included small shade tolerant herbs such as *C*. *canadensis*, *C*. *uniflora*, twinflower (*Linnaea borealis*), and five-leaved bramble (*Rubus pedatus*).
***PFT 8****—Shade tolerant ericaceous shrubs*. Cover of PFT 8 increased over time in all zones, particularly in the ESSF where by year 20 it had 36.9% cover compared to 6.0% in the ICH and 4.6% in the SBS. Characteristic species included *V*. *membranaceum*, false azalea (*Menziesia ferruginea*), and white-flowered rhododendron (*Rhododendron albiflorum*).

### Floristic response to clearcutting and slashburning

**Fig 4:**
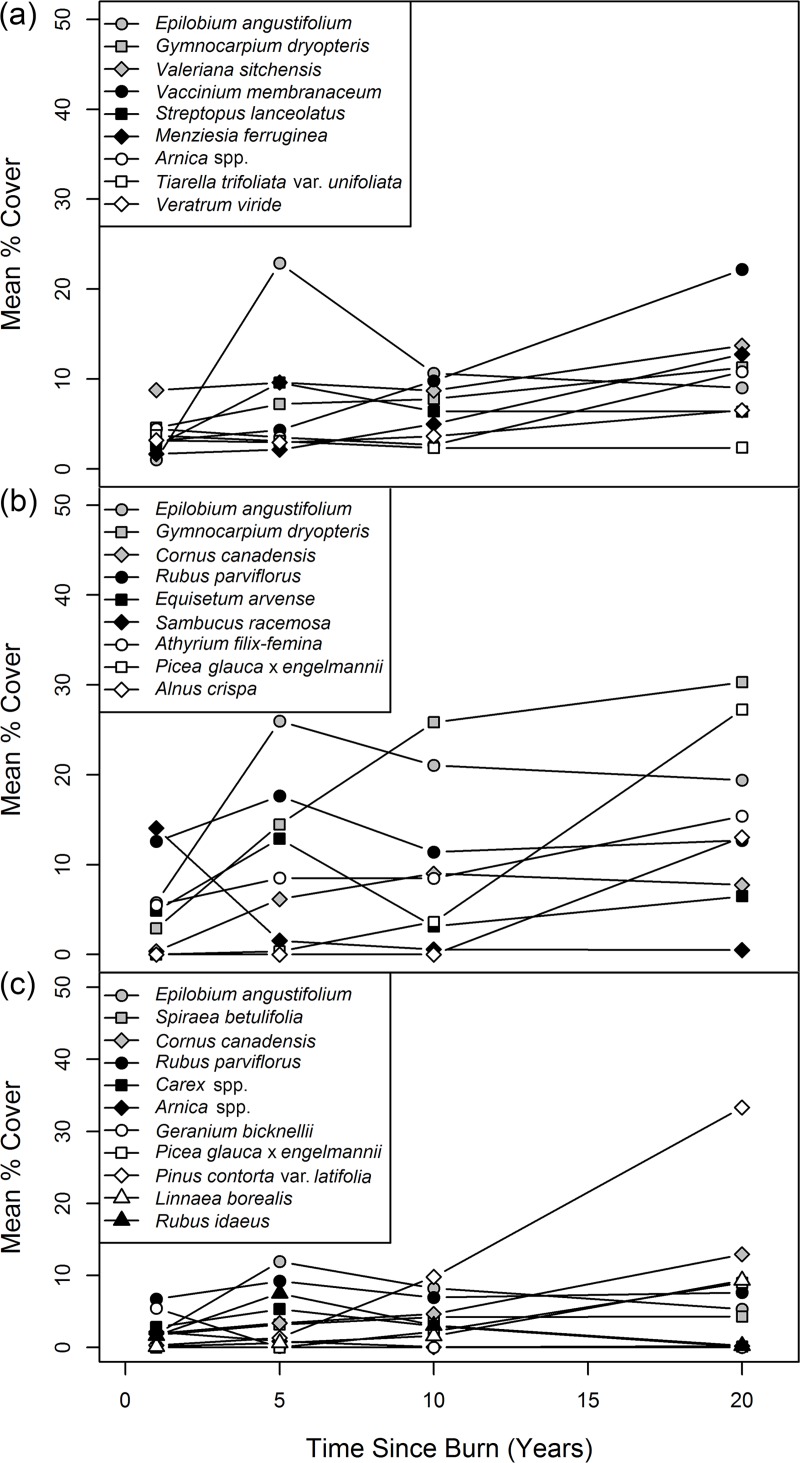
Mean percent cover of the five most abundant species in measurement years 1, 5, 10, and 20 in the ESSF (a), ICH (b) and SBS (c) zones.

Species richness increased in all zones (ESSF, *n* = 2; ICH, *n* = 2; and SBS, *n* = 6) between the first year after burning (44, 39, and 58 species respectively) and the end of the monitoring period approximately 20 years later (61, 76, 101 species respectively). For all zones combined, richness increased from 79 species in year 1 to 132 species 20 years after clearcutting and slashburning. The five most abundant species (mean % cover) in measurement years 1, 5, 10, and 20 varied by zone and over time (Fig 4); and total cover at year 20 was highest in the ICH (133%), followed by the ESSF (95%), then the SBS (82%). Furthermore, the proportion of the total cover of PFTs characteristic of mature conifer forests (i.e. low light requiring PFTs 1, 6, 7, and 8; light index < 0.5) increased over time in each zone (S3 Table, S2 Fig).

### Diagnostic species

In the ESSF there was limited cover (< 1%) of *A*. *lasiocarpa* (the only planted tree species diagnostic for this zone) by year 20 post-treatment (Table 4). All of the diagnostic understory species were present in the 20-year-old stands; cover of *Arnica* spp. and *V*. *sitchensis* was higher while cover of *R*. *pedatus* and *R*. *albiflorum* was lower than typically found in mature forests.

In the ICH zone most (4/6) of the diagnostic trees were observed in the 20-year-old stands, although with much lower cover than in the mature forest. The planted *P*. *glauca* x *engelmannii* was dominant, with very minor amounts of *T*. *plicata*, *T*. *heterophylla* (both diagnostic for this zone) and limited amounts of *A*. *lasiocarpa* (which is more characteristic of the ESSF). There was no record of *P*. *contorta* var. *latifolia* or *P*. *menziesii* var. *glauca* in the plots post burn. All diagnostic understory species for this zone were present by year 20 in one of the two stands—with lower cover of *C*. *uniflora* and *V*. *membranaceum*, and higher cover of *C*. *canadensis*, *R*. *pedatus* and *R*. *parviflorus* than in mature ICH forests.

In the SBS zone all three diagnostic trees were observed in the 20-year-old stands although *A*. *lasiocarpa* had < 0.1% cover rather than 5.1–10% typical of mature forests. Of the two planted tree species, *P*. *glauca* x *engelmannii* had cover typical of mature SBS forests, while *P*. *contorta* var. *latifolia* cover was greater than typical mature SBS forests. All of the diagnostic understory species were present on at least three of the six 20-year-old stands, except for red elderberry (*Sambucus racemosa*) and *R*. *pedatus* which were present on two of the six sites.

**Table 4:** Diagnostic species in mature forests and 20-year-old stands in ICH, SBS and ESSF zones.

		ESSF			ICH			SBS		
		*Mature forest*	*20-year-old stand*		*Mature forest*	*20-year-old stand*		*Mature forest*	*20-year-old stand*	
Species^1^	PFT^2^	Cover^3^	Cover	Constancy^4^	Cover	Cover	Constancy	Cover	Cover	Constancy
*Abies lasiocarpa*	1	25–100	0.1–1	2/2	5–10	<0.1	2/2	5–10	<0.1	4/6
*Picea engelmannii*	1	10–25								
*Picea glauca* x *engelmannii*	1		1–5	1/2	1–5	25–100	2/2	10–25	10–25	6/6
*Pinus contorta* var. *latifolia*	1		1–5	2/2	0.1–1			10–25	25–100	4/6
*Pseudotsuga menziesii* var. *glauca*	1				5–10				0.1–1	1/6
*Thuja plicata*	1				10–25	0.1–1	2/2			
*Tsuga heterophylla*	1				10–25	<0.1	1/2			
*Arnica* spp.*	2	1–5	5–10	2/2		0.1–1	2/2		0.1–1	4/6
*Lonicera involucrata*	2					<0.1	2/2	1–5	1–5	4/6
*Maianthemum racemosum*	2				0.1–1	0.1–1	1/2	∙	0.1–1	3/6
*Rubus parviflorus*	2		1–5	1/2	1–5	10–25	2/2	1–5	5–10	6/6
*Sambucus racemosa*	2		0.1–1	2/2	1–5	0.1–1	2/2	1–5	0.1–1	2/6
*Valeriana sitchensis*	2	1–5	10–25	2/2		0.1–1	1/2			
*Viburnum edule*	2							1–5	1–5	3/6
*Clintonia uniflora*	7		1–5	1/2	1–5	<0.1	1/2	1–5	1–5	5/6
*Cornus canadensis*	7		5–10	1/2	1–5	10–25	2/2	10–25	10–25	6/6
*Rubus pedatus*	7	5–10	0.1–1	2/2	1–5	10–25	2/2	1–5	0.1–1	2/6
*Rhododendron albiflorum*	8	10–25	5–10	1/2						
*Vaccinium membranaceum*	8	10–25	10–25	2/2	5–10	0.1–1	2/2	1–5	1–5	3/6

^1^ Diagnostic species (trees, shrubs and herbs, not including mosses or lichens) are those that differentiate between ecosystems at various levels in the BEC hierarchy [41].

^2^ PFT (Fig 2, S1 Fig).

^3^ The original cover scale for mature forest diagnostic species [41] begins with category 0.1%-1%. We added a category of < 0.1%. Categories 1.1%-5%, 5.1%-10%, 10.1%-25%, and 25.1%-100% appear in the table as 1–5, 5–10, 10–25, and 25–100 respectively.

4 The number of study sites in which the species was recorded. The number of study sites varied by BEC zone: ICH = 2, SBS = 6, ESSF = 2.

* *Arnica latifolia* was the original diagnostic species used for the ESSF [41]. We have combined *Arnica latifolia* with *Arnica cordifolia* as it is known that these two species hybridize readily.

### Indicator species

The ESSF had the strongest indicator species in any year measured (S4 Table). *V*. *membranaceum* and *V*. *sitchensis* were strong indicators showing almost complete specificity and fidelity over the whole 20 year time period (Table 5). *M*. *ferruginea* and brewer’s miterwort (*Mitella breweri*) were strong indicators in years 5 through 20, with *M*. *breweri* having complete specificity in all years. Strong indicators for the ICH changed between year 1, 5 and 10 with *S*. *racemosa* in year 1, trailing black currant (*Ribes laxiflorum*) and common horsetail (*Equisetum arvense*) in year 5, and three-leaved foamflower (*Tiarella trifoliata* var. *trifoliata*) and oak fern (*Gymnocarpium dryopteris*) (which also became the dominant species) in years 10 and 20. Devil’s club (*Oplopanax horridus*) was the only indicator with nearly complete specificity (> 0.9) from year 1 to year 20; it was also the only indicator in the ICH to not change in cover. Strong indicators in the SBS also changed over time with Bicknell's geranium (*Geranium bicknellii*) and *C*. *canadensis* in year 1, no strong indicators in year 5, *P*. *contorta* var. *latifolia* in years 10 and 20 (also the dominant species), and *S*. *betulifolia* in year 20.

**Table 5:** Indicator species abundance over time. Repeated measurements of indicator species percent cover 1, 5, 10, and 20 years after clearcutting and slashburning in ESSF (2 sites; *n* = 44 plots), ICH (2 sites; *n* = 136 plots), and SBS (6 sites; *n* = 49 plots) zones. Bold values indicate species with indicator values > 0.75 for the specified year; underlined values indicate that this species was not an indicator in this year.

		Year 1			Year 5			Year 10			Year 20			Friedman chi-squared	
	PFT	mean	se	^* *^	mean	se	^* *^	mean	se	^* *^	mean	se	^* *^		*p*-value
**ESSF**															
*Anaphalis margaritacea*	3	0.0	(0.0)	^*a*^	0.5	(0.1)	^*ab*^	1.1	(0.2)	^*bc*^	3.1	(0.8)	^*c*^	48.3	<0.001
*Arnica* spp.	2	4.4	(1.0)	^*a*^	**3.3**	(0.6)	^*a*^	**2.4**	(0.5)	^*a*^	3.8	(2.2)	^*a*^	1.5	0.684
*Clintonia uniflora*	7	0.3	(0.1)	^*a*^	2.0	(0.6)	^*ab*^	1.8	(0.5)	^*b*^	2.5	(0.6)	^*b*^	26.4	<0.001
*Gymnocarpium dryopteris*	2	4.6	(1.3)	^*a*^	7.2	(1.8)	^*b*^	7.8	(1.6)	^*b*^	11.3	(2.0)	^*b*^	37.0	<0.001
*Hieracium albiflorum*	3	0.0	(0.0)	^*a*^	0.4	(0.1)	^*b*^	0.5	(0.1)	^*b*^	1.1	(0.3)	^*b*^	39.0	<0.001
*Luzula parviflora*	3	0.4	(0.2)	^*a*^	0.6	(0.2)	^*ab*^	**0.5**	(0.1)	^*b*^	0.6	(0.2)	^*ab*^	19.2	<0.001
*Menziesia ferruginea*	8	1.7	(0.5)	^*a*^	**2.1**	(0.5)	^*a*^	**5.0**	(0.8)	^*b*^	**12.8**	(1.7)	^*c*^	73.7	<0.001
*Mitella breweri*	2	0.9	(0.2)	^*a*^	**2.6**	(0.6)	^*b*^	**2.6**	(0.5)	^*b*^	**2.7**	(1.2)	^*ab*^	31.0	<0.001
*Rhododendron albiflorum*	8	0.9	(0.5)	^*a*^	1.4	(0.6)	^*a*^	2.7	(0.9)	^*ab*^	6.3	(2.0)	^*b*^	42.5	<0.001
*Senecio triangularis*	2	0.0	(0.0)	^*a*^	0.2	(0.1)	^*a*^	0.2	(0.1)	^*a*^	0.8	(0.3)	^*a*^	14.9	0.002
*Streptopus lanceolatus*	2	**2.6**	(0.5)	^*a*^	**9.6**	(1.5)	^*b*^	6.4	(0.9)	^*b*^	6.4	(1.0)	^*b*^	37.8	<0.001
*Tiarella trifoliata* var. *unifoliata*	2	**3.7**	(0.6)	^*a*^	3.1	(0.5)	^*ab*^	2.3	(0.4)	^*ab*^	2.4	(0.5)	^*b*^	14.6	0.002
*Vaccinium membranaceum*	8	**3.1**	(0.7)	^*a*^	**4.3**	(0.7)	^*a*^	**9.8**	(1.6)	^*b*^	**22.2**	(1.9)	^*c*^	99.8	<0.001
*Vaccinium ovalifolium*	8	1.2	(0.4)	^*a*^	1.9	(0.4)	^*ab*^	2.8	(0.5)	^*bc*^	7.8	(1.5)	^*c*^	44.1	<0.001
*Valeriana sitchensis*	2	**8.8**	(2.0)	^*a*^	**9.6**	(2.0)	^*ab*^	**8.7**	(1.5)	^*ab*^	**13.7**	(2.3)	^*b*^	11.3	0.010
*Veratrum viride*	2	3.2	(0.9)	^*a*^	2.9	(0.6)	^*a*^	3.6	(0.8)	^*ab*^	6.5	(1.1)	^*b*^	22.3	<0.001
*Viola* spp.	2	0.9	(0.2)	^*a*^	1.3	(0.4)	^*ab*^	1.2	(0.3)	^*ab*^	2.4	(0.6)	^*b*^	17.4	<0.001
**ICH**															
*Athyrium filix-femina*	2	5.5	(1.3)	^*a*^	8.5	(1.5)	^*b*^	8.5	(1.4)	^*bc*^	15.4	(2.1)	^*c*^	86.9	<0.001
*Dryopteris expansa*	2	0.8	(0.2)	^*a*^	1.9	(0.7)	^*ab*^	3.6	(0.8)	^*b*^	2.6	(0.6)	^*ab*^	36.0	<0.001
*Epilobium angustifolium*	2	5.8	(0.7)	^*a*^	26.0	(1.6)	^*b*^	21.0	(1.8)	^*c*^	19.4	(1.6)	^*c*^	124.3	<0.001
*Epilobium ciliatum*	3	0.9	(0.2)	^*a*^	0.2	(0.1)	^*b*^	0.0	(0.0)	^*b*^	0.0	(0.0)	^*b*^	181.5	<0.001
*Equisetum arvense*	2	4.9	(0.9)	^*a*^	**12.9**	(1.8)	^*b*^	3.1	(0.6)	^*a*^	6.5	(1.1)	^*c*^	72.8	<0.001
*Galium triflorum*	2	0.1	(0.0)	^*a*^	0.9	(0.3)	^*b*^	0.3	(0.1)	^*b*^	0.4	(0.1)	^*ab*^	28.5	<0.001
*Gymnocarpium dryopteris*	2	2.9	(0.8)	^*a*^	14.5	(1.7)	^*b*^	**25.8**	(2.0)	^*c*^	**30.3**	(2.2)	^*c*^	203.2	<0.001
*Oplopanax horridus*	2	2.0	(0.5)	^*a*^	2.6	(0.7)	^*a*^	2.2	(0.5)	^*a*^	3.3	(0.9)	^*a*^	3.7	0.298
*Picea glauca* x *engelmannii*	1	0.0	(0.0)	^*a*^	0.3	(0.1)	^*ab*^	3.6	(0.8)	^*b*^	27.2	(2.7)	^*c*^	244.1	<0.001
*Ribes laxiflorum*	3	2.1	(0.3)	^*a*^	**3.1**	(0.4)	^*b*^	0.9	(0.1)	^*c*^	0.8	(0.2)	^*c*^	130.9	<0.001
*Rubus parviflorus*	2	12.6	(1.7)	^*a*^	17.6	(1.8)	^*b*^	11.4	(1.2)	^*a*^	12.7	(1.2)	^*a*^	30.0	<0.001
*Sambucus racemosa*	2	**14.1**	(1.8)	^*a*^	1.5	(0.2)	^*b*^	0.6	(0.1)	^*c*^	0.5	(0.2)	^*c*^	236.5	<0.001
*Streptopus lanceolatus*	2	0.3	(0.1)	^*a*^	1.8	(0.4)	^*b*^	3.1	(0.5)	^*b*^	7.3	(0.9)	^*c*^	121.3	<0.001
*Tiarella trifoliata* var. *trifoliata*	2	1.8	(0.3)	^*a*^	2.8	(0.5)	^*b*^	**3.0**	(0.5)	^*bc*^	**3.7**	(0.4)	^*c*^	46.3	<0.001
**SBS**															
*Betula papyrifera*	4	0.0	(0.0)	^*a*^	0.5	(0.2)	^*a*^	0.8	(0.3)	^*a*^	3.4	(1.3)	^*a*^	17.9	0.000
*Carex* spp.	3	2.8	(1.3)	^*ab*^	5.3	(1.8)	^*b*^	3.0	(1.5)	^*a*^	0.1	(0.1)	^*a*^	47.3	<0.001
*Cornus canadensis*	7	**1.9**	(0.3)	^*a*^	3.4	(0.6)	^*a*^	4.7	(0.7)	^*a*^	12.9	(2.2)	^*b*^	60.5	<0.001
*Corydalis sempervirens*	3	0.6	(0.2)	^*a*^	0.0	(0.0)	^*a*^	0.0	(0.0)	^*a*^	0.0	(0.0)	^*a*^	45.0	<0.001
*Geranium bicknellii*	3	**5.4**	(1.1)	^*a*^	0.0	(0.0)	^*b*^	0.0	(0.0)	^*b*^	0.0	(0.0)	^*b*^	93.9	<0.001
*Hieracium scouleri*	3	0.0	(0.0)	^*a*^	0.0	(0.0)	^*a*^	0.2	(0.1)	^*ab*^	0.5	(0.2)	^*b*^	43.5	<0.001
*Linnaea borealis*	7	0.1	(0.1)	^*a*^	0.6	(0.2)	^*a*^	1.6	(0.6)	^*a*^	9.3	(2.2)	^*b*^	67.2	<0.001
*Paxistima myrsinites*	7	0.1	(0.0)	^*a*^	0.2	(0.1)	^*a*^	0.4	(0.1)	^*a*^	1.4	(0.5)	^*a*^	27.4	<0.001
*Pinus contorta* var. *latifolia*	1	0.3	(0.1)	^*a*^	1.3	(0.2)	^*a*^	**9.8**	(1.5)	^*b*^	**33.3**	(3.4)	^*c*^	105.8	<0.001
*Populus tremuloides*	4	0.3	(0.1)	^*a*^	0.4	(0.2)	^*a*^	2.1	(0.9)	^*a*^	3.3	(1.1)	^*a*^	24.4	<0.001
*Ribes glandulosum*	2	0.2	(0.1)	^*a*^	0.6	(0.2)	^*a*^	0.4	(0.2)	^*a*^	0.1	(0.1)	^*a*^	14.6	0.002
*Rosa acicularis*	2	0.3	(0.1)	^*a*^	1.0	(0.4)	^*a*^	1.2	(0.5)	^*a*^	0.8	(0.3)	^*a*^	12.8	0.005
*Rubus idaeus*	3	1.6	(0.4)	^*a*^	7.5	(1.3)	^*b*^	3.1	(0.6)	^*b*^	0.2	(0.1)	^*a*^	69.5	<0.001
*Spiraea betulifolia*	2	1.7	(0.5)	^*a*^	3.2	(1.1)	^*ab*^	4.2	(1.0)	^*bc*^	**4.3**	(1.0)	^*c*^	36.4	<0.001
*Taraxacum officinale*	3	0.0	(0.0)	^*a*^	0.0	(0.0)	^*a*^	0.1	(0.0)	^*ab*^	0.6	(0.2)	^*b*^	40.0	<0.001

## Discussion

### PFTs

Development of PFTs was an effective method of data reduction in terms of the number of functional traits, and useful in generalizing community response to disturbance. Cover of the semi-shade tolerant gap specialists (PFT 2) increased in all zones between year 1 and year 20. This indicates that by year 20 in all zones, the canopy had yet to close completely and light was sufficient to support plants with relatively high light requirements (S3 Table).

Cover of PFT 3 (gap specialists with the highest light requirement; S3 Table) declined in the ICH and SBS after year 5, indicating some amount of canopy closure fairly early in the regeneration stage. In contrast, PFT 3 cover in the ESSF continued to increase over the 20 year measurement period. The earlier trajectory towards a closed canopy in the ICH and SBS suggests that these ecosystems are more resilient than the ESSF. However, this interpretation is complicated by the fact that even mature ESSF forests are considerably open [71, 40] with higher ground level light availability compared to the other 2 zones, therefore, shade intolerant species would be expected to persist longer in that zone. Compared to the other zones, the SBS had the greatest PFT 3 cover the first and fifth year post-burn, (approximately 16%; Table 2), but also had the greatest proportional decline (80%) by year 20 (S2 Fig), while the ICH had the lowest cover of PFT 3 by year 20 (Table 3).

### Floristic response to clearcutting and slashburning

Five years post-burn, all three zones developed into open meadows with fireweed (*Epilobium angustifolium*) as the dominant species with a mixture of *V*. *sitchensis* in the ESSF; *R*. *parviflorus* and *G*. *dryopteris* in the ICH; and *R*. *parviflorus* and red raspberry (*Rubus idaeus*) in the SBS. The early dominance of *E*. *angustifolium* after clearcut and/or fire is consistent with previous studies [10, 13, 38–40, 72]; however, in all three zones, *E*. *angustifolium* began to decline after year 5 and was no longer the dominant species (mean % cover < 20%) by year 20.

The vegetation community in the open young forests in the ESSF seemed to resemble that supported prior to burning [46] with *V*. *membranaceum*—*M*. *ferruginea*—*V*. *sitchensis* dominated understory (Fig 4A). Similarly, in the ICH and the SBS, forbs with limited lateral spread (*C*. *canadensis* and *L*. *borealis*), rapid lateral spread (*G*. *dryopteris*), and budbank and seedbank (*R*. *parviflorus*) regeneration strategies [73] appeared to return [47, 49–52]. However, even by year 20, residual *V*. *membranaceum* (in the ICH and SBS) and two forbs with limited lateral spread (*R*. *pedatus* in the ICH and *C*. *uniflora* in the SBS) did not reestablish as significant components of the understory community (Fig 4B and 4C).

### Diagnostic species

For the most part, understory diagnostic species found on these sites were more diagnostic of zones than were the conifer tree species (PFT 1). This is because trees were planted, while all other species regenerated naturally. The planted tree species are typically species that are well adapted to the site and grow rapidly in the conditions found in the early stages of forest development (i.e., high light levels, variable moisture conditions, competing vegetation) and not necessarily the diagnostic tree species which are typical of the old growth stage sampled in the development of the BEC system. Moreover, although we know which species were planted on each site, we don’t know how much of the cover of a species is attributed to a planted tree versus a naturally regenerated one. Therefore, results are confounded by the fact that the trees evident at year 20 are a combination of planted and naturally regenerated species.

The presence of species in PFTs 2, 7 and 8 as diagnostic species speaks to their ability to tolerate conditions found in the understory of these forests, i.e. low light levels, cool moist soils, and thick LFH layers. Our 20-year post-treatment results demonstrate their ability to tolerate the disturbances that these forests have experienced and re-establish either through resprouting or establishing from banked or transported seed [73]. Conversely, species that are less able to persist in these disturbed environments, such as shade intolerant herbs (PFT 3) and shade intolerant deciduous trees and shrubs (PFT 4) are generally not diagnostic species.

In the ESSF, the study sites we sampled shifted from forests dominated by *P*. *engelmannii* and *A*. *lasiocarpa* to stands consisting of limited amounts of planted tree species (*P*. *glauca* x *engelmannii* and *P*. *contorta* var. *latifolia* with < 5% cover; *A*. *lasiocarpa* with < 1% cover). In the ICH the composition of the canopy layer in the newly developing forests will potentially be significantly different from the previous forests—as indicated by the limited amount (< 1% cover) of diagnostic species including *T*. *plicata*, *T*. *heterophylla* and *P*. *menziesii* var. *glauca*. These species may seed in over time from adjacent mature forest stands, if there are such forests in sufficiently close proximity; otherwise the new forests will likely be dominated by *P*. *glauca* x *engelmannii*, the planted tree species. SBS overstory tree species in the young forests are more similar to those typical of the mature forest than is the case in the ESSF and ICH. *P*. *glauca* x *engelmannii* and *P*. *contorta* var. *latifolia*, both diagnostic of the mature forest in the SBS, are the dominant trees in the SBS study sites with 33% cover by year 20 (Table 5).

All understory diagnostic species for all three zones were growing in the 20-year-old stands (Table 4). This suggests that these forests are fairly resilient, at least in terms of the diagnostic species. These species have been able to re-establish, demonstrating that the sites have not been altered by the forest management treatments (i.e. clearcutting, burning and planting) to the extent that precludes a return of species that are well adapted to those sites.

### Indicator species

All diagnostic species in the ESSF were indicator species with the exception of *R*. *pedatus* (PFT 7; < 1% cover by year 20). Moreover, the four indicator ericaceous shrubs (PFT 8) of the ESSF (*V*. *membranaceum*, *M*. *ferruginea*, *R*. *albiflorum*, and oval-leaved blueberry (*Vaccinium ovalifolium*)) were indicators in all measurement years, and provided nearly half the cover of the entire set of 15 indicator species at year 20. Although *R*. *albiflorum* was a diagnostic species, it had the lowest cover of PFT 8 indicators in all years measured (6% cover at year 20) suggesting that while it is also residual with a similar regeneration strategy [73] and rooting depth, its sprouting is less vigorous (S2 Table) and it may take longer to recover from the forest treatments applied to these sites.

In the ICH, *P*. *glauca* x *engelmannii* became an indicator at year 20, compared to the ESSF which had no indicator from PFT 1, and the SBS which had *P*. *contorta* var. *latifolia* as an indicator from year 1 onwards (and as a strong indicator in years 10 and 20). All understory indicator species in the ICH found 20 years post-treatment were grouped into PFT 2, and between year 5 and 10, *G*. *dryopteris* (which is shade-tolerant with high seed quantity, seed dispersal and seed longevity; S2 Table) became the dominant indicator species. Prior to treatment, the ICH sites supported *O*. *horridus*, a plant of high cultural significance [74, 75], however, recovery of this species appeared very slow with a little over 1% increase in cover over the entire 20 year period post-treatment to a maximum of approximately 3%.

In the SBS, *P*. *contorta* var. *latifolia* was a strong indicator in years 10 and 20, increasing 3 times in cover between these years. The closing of the canopy is suggested by the reduction of light-requiring species (PFT 3) after year 5 and the increase of deep shade specialists (PFT 7) after year 10 (Table 2). Indicator species that are considered ‘invaders’ [73] and had likely established from buried seed (four from PFT 3, and one from each of PFT 2 and 4) were no longer indictors by year 20. The presence of *L*. *borealis* and *C*. *canadensis* (PFT 7) as indicator species, which increased in cover significantly between years 10 and 20, suggests the development of environmental conditions more typical of mature conifer forest [23].

### Conclusions and management implications

Our results show how the vegetation community in three forest ecosystems responded over a 20 year period after clearcutting, slashburning, and planting. The development of PFTs provided a useful method for generalizing patterns of vascular plant response at the community level. At both PFT and taxonomic levels, loss of compositional or functional diversity was not detected suggesting that montane and subalpine forest in BC are resilient to the standard forest treatment studied herein.

This study demonstrates the difficulty in determining resilience when considering multiple metrics and with such a short window of study. When using species composition with diagnostic species it seems that the ESSF was more resilient, when examining PFTs at the ecosystem level it would appear that the ICH was more resilient, and when using indicator species with species abundance it seems that the SBS was more resilient. However, future observations may answer the following questions: will the ericaceous shrubs remain dominant, will the shade intolerant understory species persist and/or will the canopy layer of the planted conifers become dominant in the colder, higher elevation ESSF; will the composition of the canopy layer diagnostic species seed in over time in the more productive ICH; and how long will it take for the deciduous trees and tall shrubs (occupying > 1/3 the canopy layer) to give way to the conifers in the SBS? Overall, this study demonstrates the need for: 1) careful consideration of meaningful indicators of resilience; and 2) additional long-term field studies that include (i) a greater number of study sites across a broader environmental gradient and (ii) measurements over a longer assessment window from which to draw conclusions about resilience.

Despite the limited timeframe and number of sites in the current study, establishing the tolerance of the diagnostic species to disturbance is validation of the robustness of the BEC system [76]. The BEC system was designed to enable users to classify a site based on species composition and soil properties—regardless of seral stage. Our results show that even by just 20 years after treatment, sites in our study area could be classified based on the presence of diagnostic species. Additionally, we produced a plant-trait dataset for 181 vascular plant species of central BC; we illustrated a system for simplifying a large plant dataset into functional groups useful in characterizing community response to disturbance; we outlined early successional stages by providing a detailed time-sequence as to when functional groups can be expected to establish, thrive or disappear; and we identified sensitive species and reliable indicators for ecosystem recovery after clearcutting, slashburning and tree planting in ESSF, SBS, and ICH forests of central BC.

## Supporting information

S1 Appendix Description of the traits in the species-traits dataset. (PDF)

S1 Fig Dendrogram of cluster analysis results. (PDF)

S2 Fig Proportion of the vegetation community by PFT. (PDF)

S1 Table Study site classification. (XLSX)

S2 Table Vascular plant species-traits dataset. (XLSX)

S3 Table Mean scores of plant traits by PFT. (XLSX)

S4 Table Indicator analysis results. (XLSX)
