# Supplementary material for: Twenty years of ecosystem response after clearcutting and slashburning in conifer forests of central British Columbia, Canada
Source: PLoS One. 2017 Feb 24;12(2):e0172667. doi: 10.1371/journal.pone.0172667 (PMC5325286; doi:10.1371/journal.pone.0172667)
Supplement: S1 Appendix — (PDF) [file pone.0172667.s001.pdf]

Description of the traits in the species-traits dataset:

1. Plant height: 0.0=graminoids, forbs, ferns and dwarf woody plants; 0.2=low shrubs and herbs; 0.4=medium shrubs; 0.6=tall shrubs; 1.0=trees [1:9].

2. Plant duration: 0=annual; 0.5=annual and/or biennial and/or perennial; 1=perennial [1:9].

3. Leaf duration: 0=deciduous (one growing season); 0.5=semi-deciduous to semi-evergreen (typically some basal leaves overwinter under snowpack); 1=evergreen [1:9].

4. Nitrogen-fixing bacteria: 0=absent; 1=present [10, 11].

5. Depth of rooting: 0.0=no roots; 0.1=very shallow-on rock or rooting in duff only; 0.3=shallow; 0.6=moderate; 1.0=deep [1:9].

6. Vigour of sprouting: 0=nonsprouting; 0.3=weak; 0.6=moderate; 1.0=strong [1:9].

7. Rate of lateral spread: 0.0=no spread; 0.3=weak-tufted plants able to get larger; 0.6=moderate-rhizomatous plants that typically become mat-forming; 1.0=rapid, wide spreading, from suckers, underground stems etc. [1:9, 12].

8. Dominant mycorrhizal guild: 1=primary; 0.5=secondary; 0=not applicable. Categories: arbuscular mycorrhizal or non-mycorrhizal; ectomycorrhizal; ericoid mycorrhizae; mixotrophic and mycoheterotrophic species with orchid mycorrhizae, ect-endo, arbutoid or a variable or uncertain combination [13].

9. Seed size: 0=spores or very small; 0.25=small; 0.5=medium; 0.75=large [14:17].

10. Seed quantity: 0=no sexual reproduction or very few; 0.25=few; 0.5=moderate; 0.75=abundant/frequent; 1=very large quantities [1:9].

11. Seed dispersal: 0.25=limited distance (includes ant dispersal); 0.5=moderate (includes seed wings and transport by larger animals via berries, hooks, etc.); 0.75=adapted for long distance travel (small seed with plumes, pappus); 1=unlimited (spores and minute seed) [1:9].

12. Seed longevity: 0.33=short (1 year or less); 0.67=medium (transient seedbank >1 year); 1=long (persistent seedbanking in soil or canopy, can appear even if plant was not present prior to disturbance) [18, 19].

13. Ground surface material: 1=primary; 0.5=secondary; 0=not applicable. Categories: Mor; Moder and Mull; exposed mineral soil; very shallow soils; surface water [1:8, 20, 21].

14. Light index: range from 0 (tolerates deep shade, intolerant of full sun) to 1 (requires full sun, does not tolerate shade) [10, 22, 23].

15. Climate: 1=primary; 0.5=secondary; 0=not applicable. Categories: alpine tundra; montane boreal; subalpine boreal; cool temperate; cool mesothermal; semi-arid [1:8, 24].

References

1. Douglas GW, Straley GB, Meidinger DV, Pojar J. Illustrated Flora of British Columbia. Volume 1: Gymnosperms and Dicotyledons (Aceraceae through Asteraceae). Victoria, Canada: B.C. Ministry of Environment, Lands & Parks and B.C. Ministry of Forests; 1998.
2. Douglas GW, Straley GB, Meidinger DV, Pojar J. Illustrated Flora of British Columbia. Volume 2: Dicotyledons (Balsaminaceae through Cucurbitaceae). Victoria, Canada: B.C. Ministry of Environment, Lands & Parks and B.C. Ministry of Forests; 1998.
3. Douglas GW, Meidinger DV, Pojar J. Illustrated Flora of British Columbia. Volume 3: Dicotyledons (Diapensiaceae through Onagraceae). Victoria, Canada: B.C. Ministry of Environment, Lands & Parks and B.C. Ministry of Forests; 1999.
4. Douglas GW, Meidinger DV, Pojar J. Illustrated Flora of British Columbia. Volume 4: Dicotyledons (Orobanchaceae through Rubiaceae). Victoria, Canada: B.C. Ministry of Environment, Lands & Parks and B.C. Ministry of Forests; 1999.
5. Douglas GW, Meidinger DV, Pojar J. Illustrated Flora of British Columbia. Volume 5: Dicotyledons (Salicaceae through Zygophyllaceae) And Pteridophytes. Victoria, Canada: B.C. Ministry of Environment, Lands & Parks and B.C. Ministry of Forests; 2000.
6. Douglas GW, Meidinger DV, Pojar J. Illustrated Flora of British Columbia, Volume 6: Monocotyledons (Acoraceae through Najadaceae). Victoria, Canada: B.C. Ministry of Environment, Lands & Parks and B.C. Ministry of Forests; 2001.
7. Douglas GW, Meidinger DV, Pojar J. Illustrated Flora of British Columbia, Volume 7: Monocotyledons (Orchidaceae through Zosteraceae). Victoria, Canada: B.C. Ministry of Environment, Lands & Parks and B.C. Ministry of Forests; 2001.
8. Douglas GW, Meidinger DV, Pojar J. Illustrated Flora of British Columbia, Volume 8: General Summary, Maps and Keys. Victoria, Canada: B.C. Ministry of Environment, Lands & Parks and B.C. Ministry of Forests; 2002.
9. Haeussler S. Prescribed fire for forest vegetation management. FRDA Memo 198. Victoria, BC: B.C. Ministry of Forests and Forestry Canada; 1991.
10. Beaudry LJ, Coupe RA, DeLong C, Pojar J. Plant Indicator Guide for Northern British Columbia: Boreal, Sub-Boreal, and Subalpine Biogeoclimatic Zones: BWBS, SBS, SBPS, and northern ESSF. Land Management Handbook 46. Victoria, B.C.: Research Branch, B.C. Ministry of Forests; 1999.
11. Beaudry LJ, Coupe RA, DeLong C, Pojar J. Plant Indicator Guide for Northern British Columbia: the Northern Portion of the MS and ICH Biogeoclimatic Zones. Technical Report 10. Victoria, BC: Research Branch, B.C. Ministry of Forests; 2003.
12. USDA Forest Service. Fire Effects Information System (FEIS). Rocky Mountain Research Station, Fire Sciences Laboratory, Missoula MT USA. 2011. Available from: http://www.feis-crs.org/feis/
13. Wang B, Qi Y-L. Phylogenetic distribution and evolution of mycorrhizas in land plants. Mycorrhiza. 2006;16: 299-363.
14. Burton CM, Burton PJ. A Manual for Growing and Using Seed from Herbaceous Plants Native to the Northern Interior of British Columbia. Smithers, British Columbia: Symbios Research & Restoration; 2003.
15. Royal Botanic Gardens Kew. Seed Information Database (SID). Version 7.1. 2016. Available from: http://data.kew.org/sid/
16. Sera B, Sery M Number and weight of seeds and reproductive strategies of herbaceous plants. Folia Geobot. 2004;39: 27-40.

17. USDA Natural Resources Conservation Service. The PLANTS Database. National Plant Data Team, Greensboro NC USA. 2011. Available from: http://plants.usda.gov
18. Lang NL, Halpern CB. The soil seed bank of a montane meadow: consequences of conifer encroachment and implications for restoration. Can J Bot. 2007;85: 557-569.
19. Thompson K, Bakker JP, Bekker RM. The soil seed banks of North West Europe: methodology, density and longevity. Cambridge, UK: Cambridge University Press; 1997.
20. Mackinnon A, Pojar R, Coupé R. Plants of Northern British Columbia. Vancouver, Canada: Lone Pine Publishing; 2005.
21. Pojar J, Mackinnon A. Plants of the Pacific Northwest Coast. Vancouver, Canada: Lone Pine Publishing; 1994.
22. Humbert L, Gagnon D, Kneeshaw D, Messier C. A shade tolerance index for common understory species of northeastern North America. Ecol Indic. 2007;7: 195-207.
23. Krajina VJ, Klinka K, Worrall J. Distribution and ecological characteristics of trees and shrubs of British Columbia. Vancouver, Canada: Faculty of Forestry, University of British Columbia; 1982.
24. Klinka K, Krajina VJ, Ceska A, Scagel AM. Indicator Plants of Coastal British Columbia. Vancouver, Canada: UBC Press; 1989.
