# Supplementary figures and images for: Twenty years of ecosystem response after clearcutting and slashburning in conifer forests of central British Columbia, Canada
Source: PLoS One. 2017 Feb 24;12(2):e0172667. doi: 10.1371/journal.pone.0172667 (PMC5325286; doi:10.1371/journal.pone.0172667)

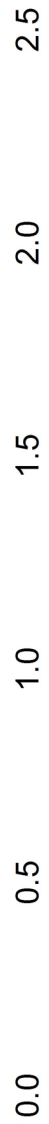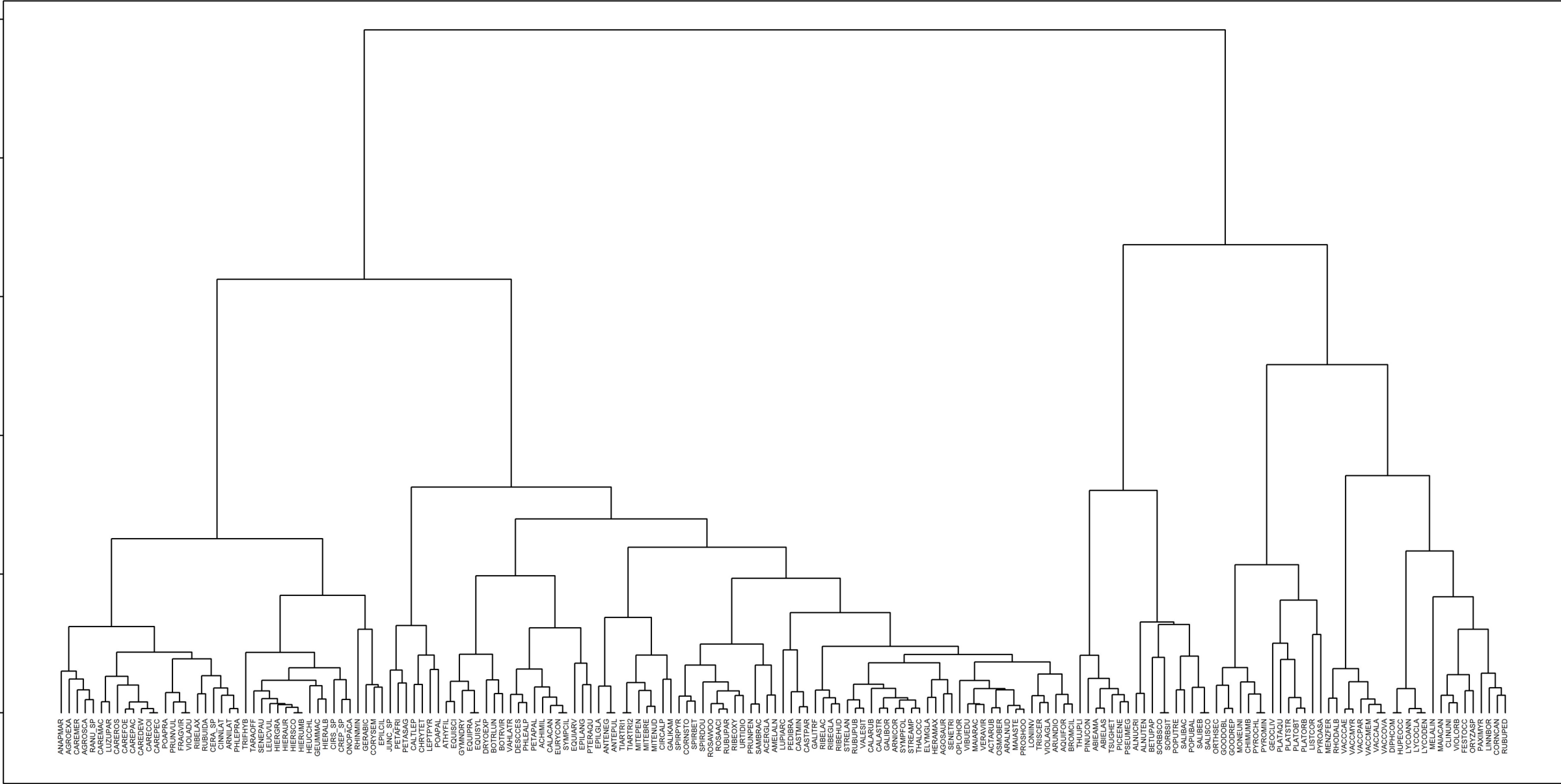

Supplement: S1 Fig — (PDF) [file pone.0172667.s002.pdf]
