# Supplementary material for: Twenty years of ecosystem response after clearcutting and slashburning in conifer forests of central British Columbia, Canada
Source: PLoS One. 2017 Feb 24;12(2):e0172667. doi: 10.1371/journal.pone.0172667 (PMC5325286; doi:10.1371/journal.pone.0172667)

Plant Functional Types in the ESSF (Year 1)

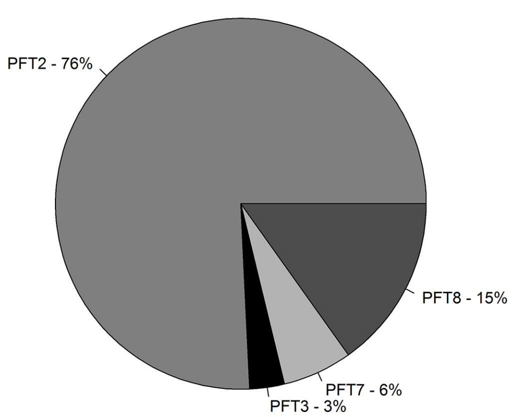

Plant Functional Types in the ESSF (Year 20)

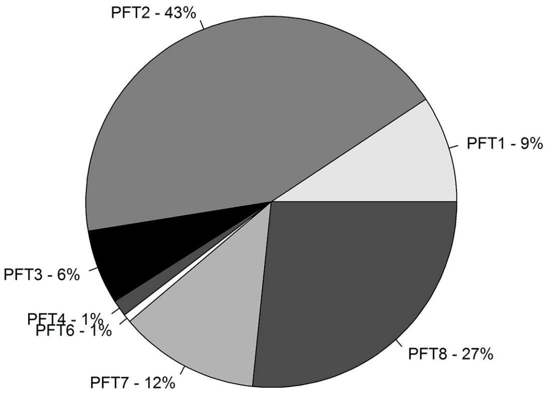

Plant Functional Types in the ICH (Year 1)

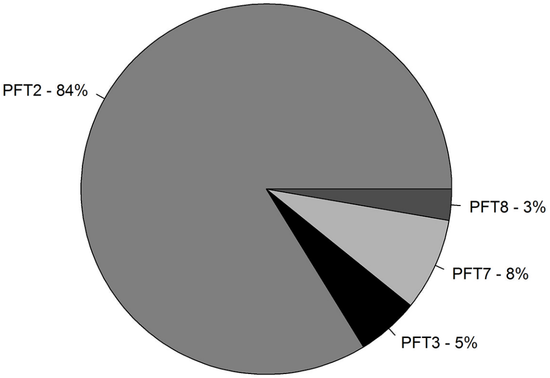

Plant Functional Types in the ICH (Year 20)

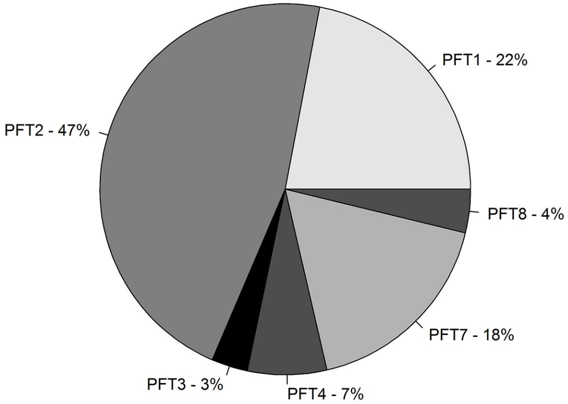

Plant Functional Types in the SBS (Year 1)

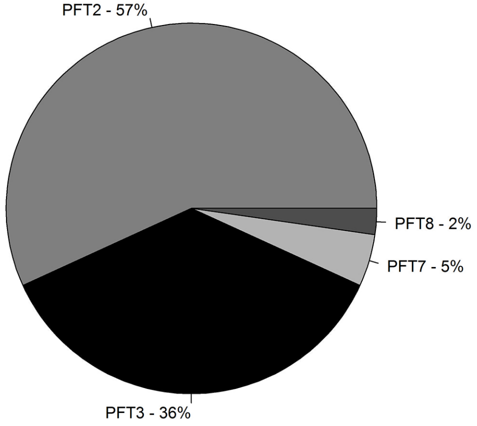

Plant Functional Types in the SBS (Year 20)

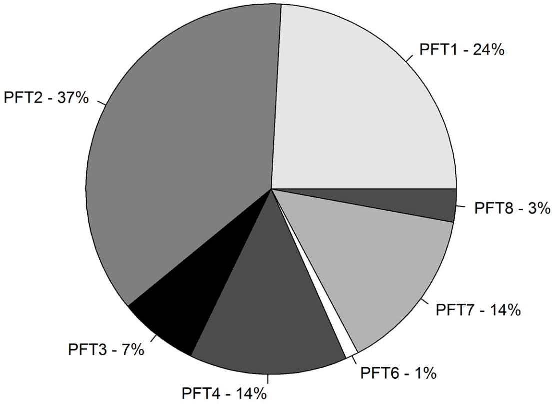

Supplement: S2 Fig — (PDF) [file pone.0172667.s003.pdf]
